# Tonsillitis from the Standpoint of the General Practitioner

**Published:** 1901-08

**Authors:** A. L. Gray

**Affiliations:** Richmond, Va., Professor of Physiology, University College of Medicine


### TONSILLITIS FROM THE STANDPOINT OF THE GENERAL PRACTITIONER.*

BY A. L. GRAY, M.D., RICHMOND, VA.,
Professor of Physiology, University College of Medicine.

There is no disease in the whole catalogue that confronts the practitioner oftener and perplexes him more than some of the forms

*Read before the Richmond Academy of Medicine and Surgery, June 25, 1901.

of acute tonsillitis. Aside from the desirability of an early diagnosis for purposes looking toward its alleviation, we are called upon frequently to make a speedy differentiation from diphtheria for reasons of prophylaxis. That the diagnosis is not easy, or even possible in the early stages in some instances, without the aid of the bacteriologist, is to my mind a positive conclusion. In order to bring out important facts, it is necessary to take up and discuss briefly the clinical varieties.

There are two forms usually described: the parenchymatous, or suppurative, and the lacunar, or follicular.

The parenchymatous or suppurative form, which is commonly known as quinsy, after some violation of hygienic laws, usually exposure to cold or wet, comes on suddenly, with perhaps one or more chills followed by fever which may reach 105° or 106° F., rapid pulse rate and great prostration. During and preceding these symptoms, the patient complains of aching in the throat and pain on swallowing. Inspection of the throat most frequently shows one side to be affected. The tonsil itself may be enlarged and reddened, but oftener the inflammation seems to exist in the peritonsillar tissue, and the whole pharynx on the affected side seems to be involved. Not infrequently involvement of the opposite side follows in rapid succession, and the space between the fauces becomes almost obliterated. In a large proportion of these cases the inflammatory process proceeds to pus formation, the abscess usually pointing toward the mouth.

This form is usually simple of diagnosis, and for this reason of less interest than the follicular or lacunar form. It is here that our skill is in some cases taxed to the utmost. Under this may be classed ulcerative, herpetic, pseudo-membranous and necrotic forms.

Follicular tonsillitis is most common in adolescence and early adult life, though the infant and those in advanced life are not exempt. I have seen it in a child of two years, and in the aged. In the latter, however, it is rare, owing to the disappearance of the adenoid tissue of the pharynx after middle life. Sex seems to have no influence, nor do I think the statement that rheumatism is a predisposing cause can be substantiated. That the disease is due to bacterial invasion is most probably the correct theory. Whether it is due to a specific organism remains yet to be absolutely determined, but there are strong reasons for this belief. How else can we explain the fact that many members of the same household will be successively attacked, or, as I have known, persons visiting a family in which the disease has attacked some member, and in the course of a few days develop an attack? Another view, and the one most commonly accepted, is that persons surrounded by the same conditions of imperfect hygiene, are rendered similarly resistant to the invasion of bacteria which are found in the throat in health, such as the streptococcus and the diplococcus lanceolatus.

Let us consider briefly the pathological condition: In each of the above varieties we find an enlarged and reddened tonsil with more or less involvement of the crypts. The most common appearance is an exudation of a yellowish-white caseous material escaping in lumps or strings from the glandular crypts. In some instances, however, instead of this material exuding, it appears to have become encysted and calcified and is not discharged until after the acute inflammation disappears and the swelling begins to subside. Not infrequently two or more adjacent crypts become distended with this material, forming, as it were, minute abscesses which rupture into each other, leaving a ragged depression or ulcer, from the bottom of which is poured this caseous exudate. Occasionally, also, patches appear in color identical with those of diphtheria, but usually not so thick; nor do they possess the same tendency to spread to the surrounding parts, but attain a certain size in the course of a day, which they maintain. These may be found not only in the tonsils, but also on the adjacent structures. They are due usually to streptococcus infection.

Another pathological condition is the appearance of herpetic vesicles which rupture and leave small white ulcers. These may appear on the tonsils and adjacent parts as well. We also sometimes find a pale, milky-white, membranous appearance on the tip and margin of the uvula and pillars of the fauces, due to the leptothrix buccalis, a normal inhabitant, which is always ready to attack an abraded surface in the mouth, or to the oidium albicans or thrush parasite.

Considering the various pathological conditions which present themselves, and the signs and symptoms based thereon, there are unquestionably cases arising in which to make a correct diagnosis without the aid of the bacteriologist is an absolute impossibility, and I am convinced that the statements of many physicians who claim to have treated diphtheria for years with scarcely a mortality without the aid of antitoxin, and even before the age of antiseptics, are erroneous in the extreme, and their glowing statistics apply not to diphtheria, but to some form of follicular tonsillitis. That a bacteriologic examination in the onset is impracticable in many cases cannot be denied, and in these our powers of differentiation are most severely taxed.

As to the modes of onset of the two diseases, they must be admitted to be in many instances identical. An initial chill or convulsion, subsequent rise of temperature in some instances to an alarming degree, with delirium accompanying, pains in the head, neck, back and joints, and great prostration, are common to both. We must look then to the throat alone to guide us in our prognosis and treatment.

The throat appearances in diphtheria, as told in most of our text-books, leave us under the impression that whenever we find a grayish-white or yellowish pseudo-membrane on the tonsils which cannot be easily removed without abrading the surface, and especially if seen on the adjacent mucous membrane, a positive diagnosis of this disease must be made. This is manifestly incorrect. In cases of tonsillitis which present an ulcerative appearance the bases of these ulcers are at first covered with a dirty-yellowish or grayish necrotic tissue which may continue firmly adherent for several days. These ulcerations are by no means always confined to the tonsils, but may appear on the posterior pharyngeal wall. The cases which simulate diphtheria most strongly are those in which we have here and there patches of distinctly superficial pseudo-membrane produced by fungi or bacteria other than the Klebs-Lœffler bacillus. These patches will generally be found discrete, and the point of most value to us is that they do not rapidly spread at their margins in an even layer, but retain their size and shape, and when due to the leptothrix buccalis, are very thin and milky-white in appearance. Whenever practicable, however, in any case in which there is a shadow of doubt, a competent bacteriologist should be called in at the earliest possible moment, lest valuable time be lost and our labors be in vain.

As regards treatment: At the onset, it is well to give a brisk saline purge, treating the febrile symptoms with one of the antipyretics. I have found phenacetine, grs. 5, codeine sulphate, gr. $\frac{1}{4}$, administered every three hours, to afford great relief from the headache; and salol in doses of three to five grains every four hours, the most satisfactory remedy for the aching joints.

In the parenchymatous form, in the early stages, the icebag or a substitute, externally, and cracked ice given the patient at short intervals, or ice cream, will be found both agreeable and beneficial. After the inflammatory process has progressed, hot poultices or fomentations will be found more soothing and more apt to reduce the inflammation. Counterirritation in the form of tincture of iodine or hot turpentine stupes will be productive of much good. Scarification of the swollen parts will give great relief; and as soon as pus be detected, it should be evacuated, preferably through the anterior surface of the soft palate, as this is the location at which the abscess usually points; and through an incision at this point, the abscess cavity can be readily cleansed without causing much discomfort to the patient.

In the follicular variety, if begun in time, ice applications, both internally and externally, may sometimes abort an attack. After it is well established, there is no better treatment than a spray of equal parts of hydrogen peroxide and water to clean off the exudate, and as a germicide, followed by a mixture of the tincture of ferric chloride and chlorate of potash in equal parts of glycerine and water, to be applied to the tonsils and surrounding inflamed surfaces, with a camel's-hair brush, four or five times a day. In the intervals, some demulcent application, as a spray of menthol or thymol in liquid vaseline, will lessen the difficulty in swallowing and be found most grateful to the patient. The ammoniated tincture of guaiacum given internally and as a gargle is most warmly recommended by many as a specific. Counterirritation externally to the throat is desirable in this form, also, and is probably best produced by rubbing with cloths sprinkled with oil of turpentine or spirits of camphor. The diet should be semi-solid or liquid, as it is swallowed with less difficulty and does not tend to keep up the inflammation by mechanical irritation. Following this line of treatment, I have seldom found the disease to continue longer than three or four days.

It is my firm belief that in the near future, follicular tonsillitis will be found to be due to specific bacterium, and that its infectiousness will be firmly established; and I think we err when we allow our cases to be closely associated for any length of time with other members of the family or household. For while it is not a grave malady so far as mortality is concerned, I know of no febrile disease that renders its victim more miserable for the time being, and I think that by isolation for a short time, we may confine the disease to a single individual and thereby avert much suffering.

901 East Clay street.

